# The Outwardly Rectifying Current of Layer 5 Neocortical Neurons that was Originally Identified as “Non-Specific Cationic” Is Essentially a Potassium Current

**DOI:** 10.1371/journal.pone.0132108

**Published:** 2015-07-21

**Authors:** Omer Revah, Lior Libman, Ilya A. Fleidervish, Michael J. Gutnick

**Affiliations:** 1 Koret School of Veterinary Medicine, Robert H. Smith Faculty of Agriculture, Food, and Environment, The Hebrew University of Jerusalem, Rehovot, Israel; 2 Department of Physiology and Cell Biology, Faculty of Health Sciences and Zlotowski Center for Neuroscience, Ben-Gurion University of the Negev, Beer Sheva, Israel; Sackler Medical School, Tel Aviv University, ISRAEL

## Abstract

In whole-cell patch clamp recordings from layer 5 neocortical neurons, blockade of voltage gated sodium and calcium channels leaves a cesium current that is outward rectifying. This current was originally identified as a “non-specific cationic current”, and subsequently it was hypothesized that it is mediated by TRP channels. In order to test this hypothesis, we used fluorescence imaging of intracellular sodium and calcium indicators, and found no evidence to suggest that it is associated with influx of either of these ions to the cell body or dendrites. Moreover, the current is still prominent in neurons from TRPC1^-/-^ and TRPC5^-/-^ mice. The effects on the current of various blocking agents, and especially its sensitivity to intracellular tetraethylammonium, suggest that it is not a non-specific cationic current, but rather that it is generated by cesium-permeable delayed rectifier potassium channels.

## Introduction

Neocortical pyramidal neurons possess a cationic current which shows pronounced outward rectification [1]. Because it entails cesium permeability, we call it I_cs_. It survives as the only ionic current following blockade of voltage-gated Na^+^ and Ca^2+^ channels [2,3]. I_cs_ was first described by Alzheimer [1], who suggested that it is a non-specific cationic current. Subsequent authors have noted the similarity between the I-V relationship of this current and that of certain TRP channel complexes [4]. Immunohistochemical evidence indicates the presence of various TRP channels in cortical neurons [4–6], and TRP channel conductance has been hypothesized to play a role in diverse normal and pathological cortical functions [7–10]. However, there has been no conclusive evidence that I_cs_ entails flux of any cations other than Cs^+^ and K^+^. We therefore set out to determine whether I_cs_ is indeed a non-specific cationic current, and whether it may be mediated by TRP channels. We now report that all available evidence indicates that the channels responsible for I_cs_ are permeable neither to Na^+^ nor to Ca^2+^, and that it is probably an example of a cesium-permeable delayed rectifying K^+^ channel.

## Methods

### Animals

All experiments were approved by the Animal Care and Use Committee of The Hebrew University of Jerusalem. Experiments were performed in coronal slices from the somatosensory cortex of wild type (CD-1) and knockout (TRPC1^-/-^, TRPC5^-/-^) [11] mice at the postnatal day 12 to 21.

### Slice preparation and maintenance

Experimental procedures were as previously reported from our laboratory [3]. Briefly: animals of either sex were deeply anesthetized with Nembutal (60 mg/kg), and killed by decapitation. Their brains were rapidly removed and placed in cold (<4°C), oxygenated (95% O2–5% CO2) artificial cerebro-spinal fluid (aCSF). Coronal slices, 300–400μm thick, from a region corresponding to the primary somatosensory cortex were cut using a vibratome (Series 1000; Pelco International, Redding, CA) and were placed in a maintenance chamber containing aCSF at room temperature. After a minimum of half an hour of recovery time they were transferred to a recording chamber.

### Patch-clamp recording

Whole-cell recordings from layer 5 neurons were either made blindly [12,13] or under infrared differential interference contrast (IR-DIC) microscopic control [14]. For blind recording, the slices were maintained in a small (300 μl) interface-type recording chamber [15]; for visually controlled recording, slices were held submerged in a chamber on a fixed stage of an Axioskop FS microscope (Carl Zeiss, Oberkochen, Germany). Currents were recorded in whole-cell configuration using an Axopatch 200A or Axopatch 200B amplifier (Molecular Devices, Foster City, CA). Patch pipettes were manufactured from thick-walled borosilicate glass capillaries (outer diameter, 1.5 mm; Hilgenberg, Malsfeld, Germany) and had resistances of 5–7 MΩ for somatic recordings. All recordings were made at 32–36°C. Command voltage protocols were generated and whole-cell data were acquired on-line with a Digidata 1320A analog-to-digital interface. Data were low-pass filtered at 2 kHz (-3 dB, four-pole Bessel filter) and digitized at 20 kHz. Leak currents were subtracted offline.

Care was taken to maintain membrane access resistance as low as possible (usually 5–8 MΩ and always less than 13 MΩ). Capacitive currents were reduced before break-in using the amplifier circuitry.

The aCSF contained the following (in mM): 124 NaCl, 3 KCl, 2 CaCl_2_, 2 MgSO_4_, 1.25 NaH_2_PO_4_, 26 NaHCO_3_ and 10 glucose, pH 7.3 at 36°C when bubbled with a 95% O_2_−5% CO_2_ mixture. In many experiments, the aCSF also contained the following channel blockers (in μM): 200 Cd^2+^, 1 Tetrodotoxin (TTX), 20 6,7-dinitroquinoxaline-2,3-dione (DNQX) and 10 Bicuculline (BMI). Unless otherwise noted, the pipette solution contained the following (in mM): 135 CsCl, 2 MgCl_2_, and 10 HEPES (cesium salt), pH 7.25.

### Imaging

Sodium and calcium imaging were performed using Sodium-binding benzofuran isophthalate (SBFI) and Oregon Green 488 BAPTA-1 (OGB1) fluorescence (respectively), excited with a high-intensity LED device [385 or 480 nm; Prizmatix, Israel]. The emission was collected with modified Olympus U-MNU2 and U-MNIBA2 filter sets (DC = 400 nm, EM = 420 nm and DC = 505 nm, EM = 530(20) nm, respectively). Changes in fluorescence were acquired using a back-illuminated 80 × 80 pixel cooled camera (NeuroCCDSMQ; RedShirt Imaging) controlled by Neuroplex software. Images were acquired at 500 frames per second. Indicator bleaching was corrected by subtracting an equivalent trace without electrical stimulation. To improve the signal-to-noise ratio of the traces, 5 to 10 trials were typically averaged.

### Data analysis

Data averaging, digital subtraction of null traces, and current peak detection were made using pClamp 9.0 (Molecular Devices). Data were fitted using Origin (OriginLab, Northampton, MA). Values are given as mean ± SE. Student’s t test was used for statistical analysis.

The cation permeability ratio was calculated by solving the Goldman-Hodgkin-Katz equation assuming that the extracellular Cs^+^ concentration and intracellular K^+^ concentrations are equal to zero and that the channel is only permeable to these ions.

$$V_{m}=\frac{RT}{F}\text{ln}\left(\frac{p_{k}{\left[K\right]}_{o}}{p_{Cs}{\left[Cs\right]}_{i}}\right)$$

$$\frac{V_{m}F}{RT}=\text{ln}\left(\frac{p_{k}{\left[K\right]}_{o}}{p_{Cs}{\left[Cs\right]}_{i}}\right)$$

$$e^{\frac{V_{m}F}{RT}}=\frac{p_{k}{\left[K\right]}_{o}}{p_{Cs}{\left[Cs\right]}_{i}}$$

$$\frac{p_{Cs}}{p_{K}}=\frac{{\left[K\right]}_{o}}{e^{\frac{V_{m}F}{RT}}{\left[Cs\right]}_{i}}$$

When *Vm (reversal potential of the current)* = -40 mV.

## Results

**Fig 1:**
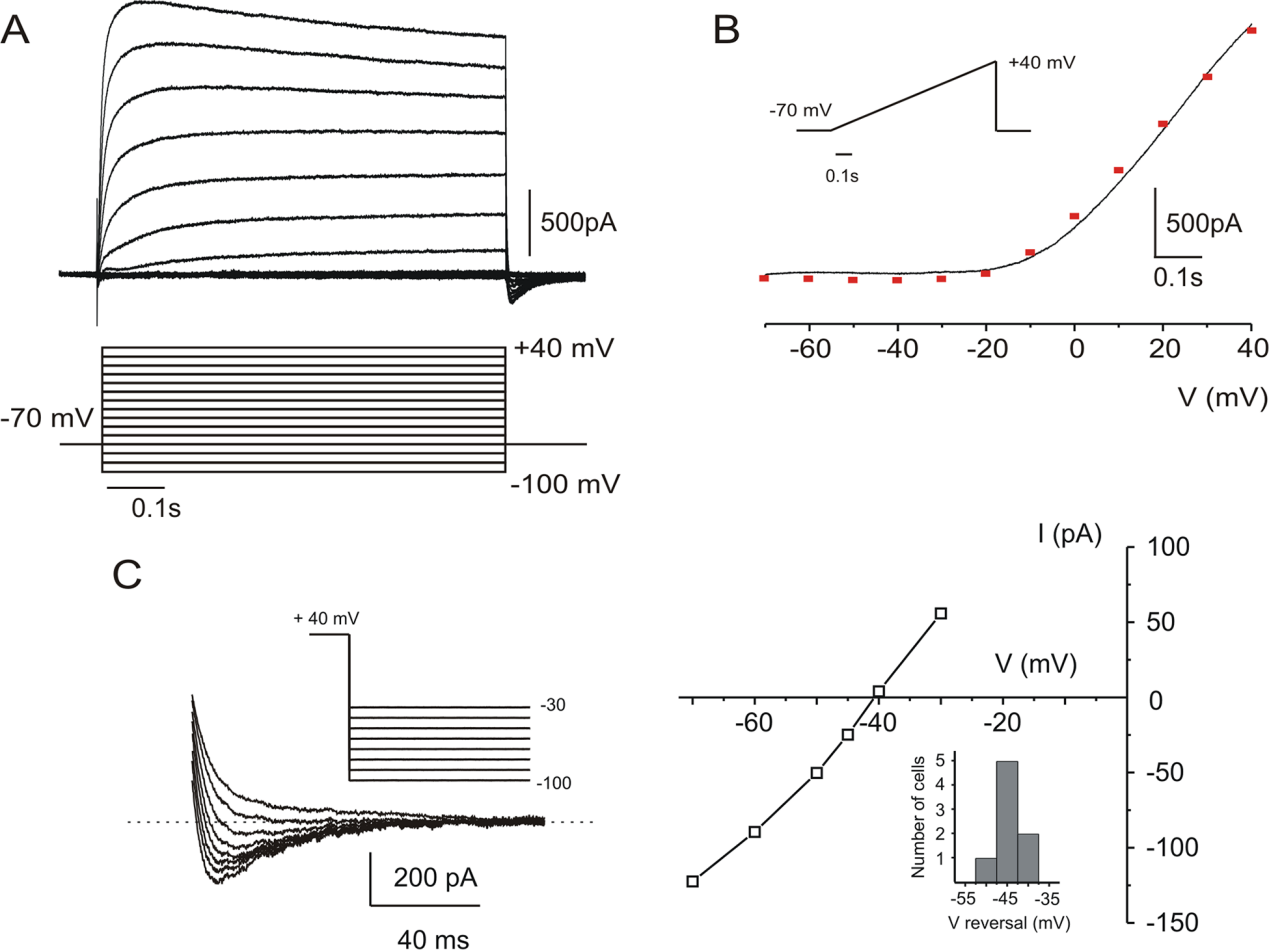
I_cs_ in cortical layer 5 pyramidal neurons. *A*: Whole cell, voltage clamp recording while blocking Na^+^ currents with TTX (1μM), Ca^2+^ currents with Cd^2+^ (200μM) and Cs^+^ (135mM) in the recording pipette to block K^+^ currents. Voltage steps (10 mV increments) revealed an outward current at voltages more depolarized than -20mV. *B*: In the same cell, a slow (110 mV/s) voltage ramp (-70 to +40 mV) generated an outward rectifying instantaneous IV curve similar to the one in A (red squares). *C*: Repolarizing voltage steps from +40 mV (10 mV increments) reveal tail currents at potentials more negative than -40 mV *(left)*. *Right*: IV curve for this cell shows reversal at around -40 mV. Inset: distribution of reversal potentials of I_cs_ for 8 neurons.

Recordings were made from 53 Layer 5 pyramidal neurons, as identified by their typical morphology, relatively large size and appropriate distance from the pia (350–550 μM). When voltage clamped, a negative current was recorded at -70 mV, as expected when Cs^+^ replaced K^+^ as the main intracellular cation. In the presence of DNQX (25 μM), APV (40 μM), BMI (10 μM), Cd^2+^ (200 μM) and TTX (1 μM), all neurons generated I_cs_: a prominent, outwardly rectifying, voltage-dependent current when depolarized to voltages more positive than -20 mV (Fig 1A and 1B). After break-in, I_**cs**_ decreased by about 15% during the first five minutes, and after this initial period of rundown, it remained stable for several hours. The peak current amplitude (+40 mV) recorded at this time was 1.93 ± 0.14 nA (n = 16 neurons). During prolonged (10 s) depolarizing voltage steps, the current underwent voltage-independent inactivation with a time constant of 6–7 seconds (data not shown).

### Voltage dependence of I_cs_

The voltage dependence of I_cs_ is shown by the family of curves in Fig 1A. The activation kinetics clearly accelerated with depolarization. Fig 1B shows the current response of the same representative cell to a slow voltage ramp from -70 to +40 mV. Both the IV curve generated by depolarizing steps (red squares) and the ramp-generated instantaneous IV curve show a prominent outward rectification. Voltage steps were followed by an inward tail current. In Fig 1C, return to different potentials after the voltage step revealed that the reversal potential of the tail was -40 mV. Alzheimer [1] suggested that the deviation of this reversal potential from -70 mV indicates that the rectifying current is carried not only by K^+^ ions (or Cs^+^ under these experimental conditions) but also by Na^+^ and/or Ca^2+^, and he therefore referred to it as a non-selective cationic current.

### Kinetics of I_cs_

**Fig 2:**
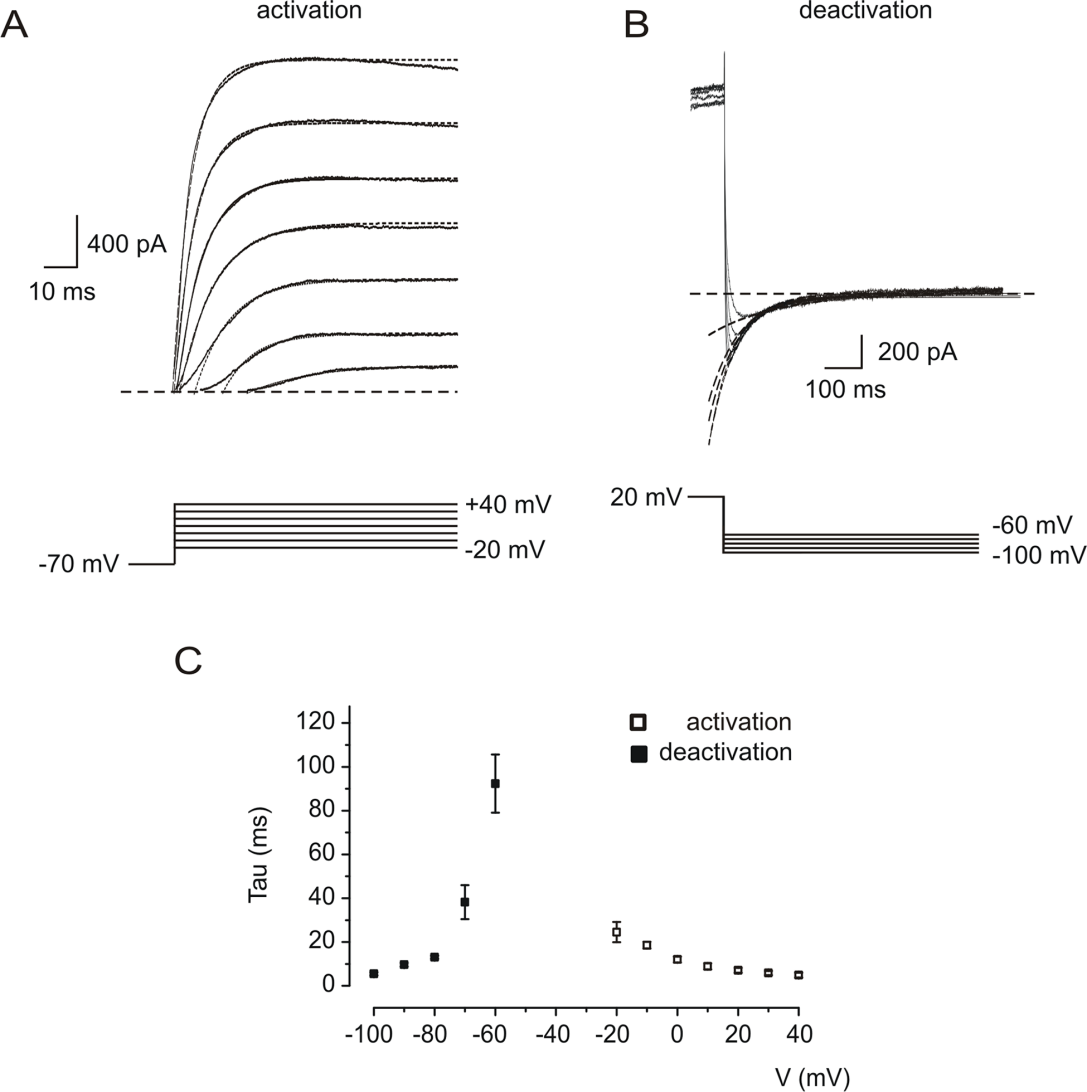
Voltage-dependent activation and deactivation of I_cs_. *A*: Response of a representative neuron to depolarizing voltage steps from a holding potential of -70 mV. Application of voltage steps (lower panel) resulted in outward currents (upper panel, solid lines) which were fitted mono-exponentially (dashed lines). *B*: Hyperpolarizing steps (lower panel) from +20 mV, resulted in inward tail currents (upper panel, solid lines). Deactivation of I_cs_ was voltage-dependent and was also fitted with single exponentials (dashed lines). *C*: Time constants of activation (empty squares) and deactivation (filled squares) as a function of membrane voltage (n = 5 neurons).

In order to study the voltage-dependence of activation of I_cs_ we applied voltage steps from -70 mV to potentials more positive than -20 mV (Fig 2A) and plotted the resultant activation time constants (Fig 2C, empty squares, n = 5). Analysis of the tail currents following steps from +20 mV to negative potentials (Fig 2B) revealed the voltage dependence of deactivation (Fig 2C, filled squares, n = 5).

### Cation permeability during I_cs_

**Fig 3:**
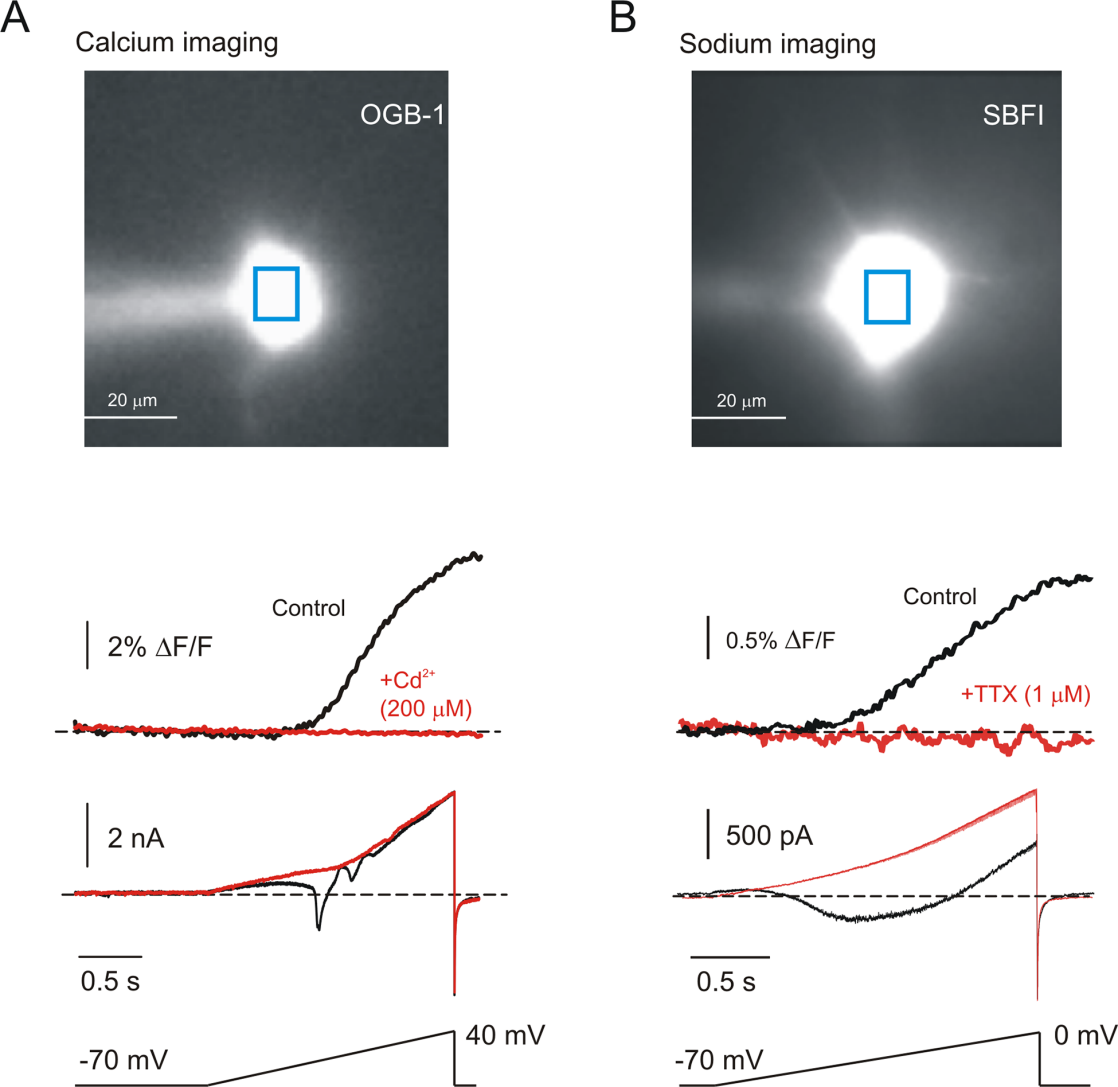
I_cs_ is not associated with detectible influx of Ca^2+^ or Na^+^. *A*: *Image*: A layer 5 neuron during fluorescent Ca^2+^ imaging with OGB-1 in the recording electrode. *Traces*: -70 to +40 mV ramps resulted in Ca^2+^ influx *(upper black trace)* and electrically recorded Ca^2+^ action currents (*middle black trace)*. Adding 200μM Cd^2+^ to the bath completely eliminated the Ca^2+^ flux (*upper red trace)*, leaving only I_cs_ (*middle red trace*). *B*: *Image*: A layer 5 neuron during fluorescent Na^+^ imaging with SBFI in the recording electrode. *Traces*: -70 to 0 mV ramps resulted in Na^+^ influx and *(upper black trace)* and electrically recorded persistent Na^+^ current (*middle black trace)*. Adding 1μM TTX completely eliminated both the Na^+^ flux and the persistent Na^+^ current.

We reasoned that if I_cs_ is indeed a non-specific cationic current, it should be associated with detectible influx of Ca^2+^ and/or Na^+^ ions. We tested this by simultaneously imaging and recording after intracellularly applying appropriate ion-selective fluorescent dyes. The cell illustrated in Fig 3A is representative of 3 neurons which were filled with OGB1 to determine Ca^2+^ flux. In the presence of 1μM TTX, a slow voltage ramp generated a sizable voltage-dependent rise in intracellular Ca^2+^ which was accompanied in the electrical trace by Ca^2+^ action currents. However, after blockade of voltage-dependent Ca^2+^ currents with Cd^2+^, all signs of Ca^2+^ flux disappeared from both the imaging and the electrical traces while I_cs_ persisted. Similarly, the cell in Fig 3B is representative of 7 cells filled with the sodium-sensitive dye, SBFI to reveal Na^+^ flux. With Cd^2+^ in the bath, a slow depolarizing ramp from -70 to 0 mV generated a large persistent Na^+^ current and a concomitant rise in intracellular Na^+^ concentration. Addition of TTX to block voltage-gated Na^+^ channels completely removed evidence of Na^+^ flux and the associated inward current, again leaving I_cs_. In both cases, these findings were true not only for the cell body but also for proximal dendrites (not shown). It is noteworthy that this imaging technique is quite sensitive to relatively small fluxes of cation in these cells. Thus, single action potentials, which are less than 1.5 ms in duration, are associated with detectible changes of somatic Na^+^ flux [16], and somatic Ca^2+^ is commonly used as an indication of action potential generation [17]. Because I_cs_ is a large, persistent current which integrates the flux, we would expect a non-negligible Na^+^ or Ca^2+^ component to be readily detected under our experimental conditions. Yet, the imaging experiments provided no evidence for a Na^+^ or a Ca^2+^ component to I_cs_.

### Pharmacological sensitivity of I_cs_

**Fig 4:**
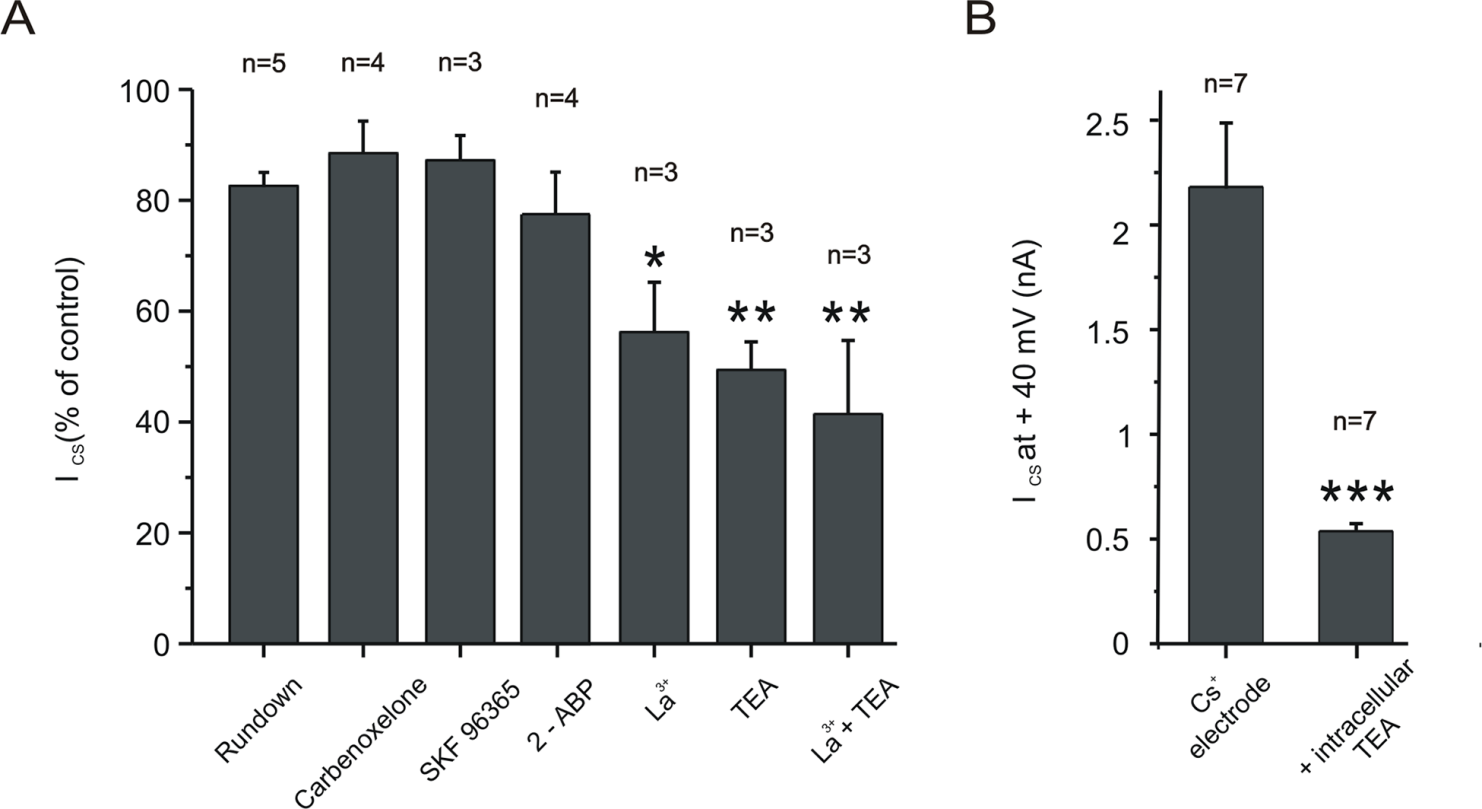
Pharmacological sensitivity of I_cs_. *A*: Changes (as compared to break-in) in current amplitude at +40 mV in response to the potential I_cs_ antagonists: carbenoxelone (100 μM), SKF96365 (100 μM), 2-APB (100 μM), La^3+^ (1 mM) and TEA (40 mM). La^3+^ concentration was reduced to 100 μM when co-applied with TEA. *B*: Comparison of current amplitudes at +40 mV when recording electrodes contained either Cs^+^ (135 mM) or Cs^+^ + TEA (30 mM of TEA replaced) to block K^+^ channels. *P* values represent comparison to control, *P* > 0.05; *P < 0.05, **P < 0.01, ***P < 0.001). Data shown are averages and error bars represent s.e.m.

We attempted to identify the molecular origin of I_cs_ by adding a variety of potential antagonists to the bathing fluid (Fig 4A). Neither the gap-junction blocker carbenoxelone (100μM) nor the pannexin1 blocker probenecid (2 mM) had any significant effect on I_cs_. We considered the possibility that I_cs_ might be mediated by one or more of the family of TRP channels, and these are notoriously difficult to manipulate pharmacologically [18]. SKF96365 (100μM), a reagent which was originally identified as a blocker of receptor-mediated Ca^2+^ entry [19], and which has been shown to be effective on several TRPC channels [20,21] also had no significant impact on I_cs_. Application of 100μM of the IP3 inhibitor, 2-ABP [22], also did not cause a significant block of the current. Application of (100μM) La^3+^, a non-specific TRP-channel blocker [23,24] did cause a significant reduction in current amplitude by 45 ± 9%. Finally, in agreement with [1], we found that extracellular application of the K^+^ channel blocker, TEA (40 mM) blocked the current by 51 ± 5% (n = 3). This, along with the imaging findings, led us to hypothesize that I_cs_ is primarily generated by K^+^ channels that are permeable to Cs^+^. TEA is specific and effective against delayed rectifier K^+^ channels when applied from the inside of the membrane [25]. Fig 4B shows that when we replaced 30 mM Cs^+^ in the intracellular pipette with TEA, I_cs_ was almost entirely blocked. The effect was quantified by comparing the current amplitude at +40 mV of cells recorded with Cs^+^ electrodes or with TEA electrodes (n = 7 neurons for each group).

### I_cs_ is present in neurons from specific TRP knockouts

**Fig 5:**
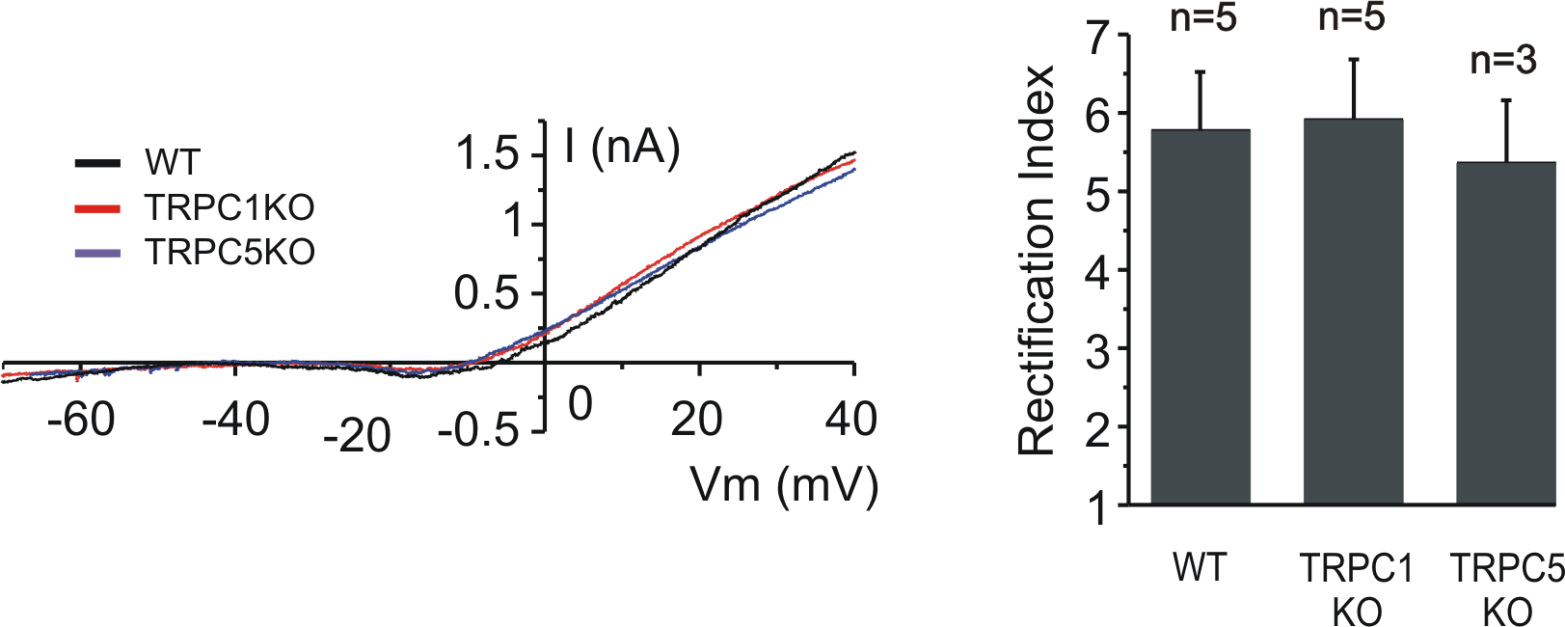
Layer 5 neurons I-V curves are similar in neurons lacking TRPC1 or TRPC5 channels. *Left*: representative instantaneous *I-V* curves during slow ramps from wild type (black), TRPC1 KO (red) or TRPC5 KO (blue). *Right*: IV curve shape for the groups, as quantified by the rectification index (see results) is not significantly different. Data shown are averages and error bars represent s.e.m. (*P* > 0.05).

Strübing et al. [4] reported that coexpression of TRPC1 and TRPC5 results in an outward rectifying non-specific cationic channel which, they suggested, might account for the current reported by Alzheimer in cortical neurons. We therefore sought I_CS_ in neurons from animals in which TRPC1 or TRPC5 had been knocked out. The representative traces in Fig 5 show that I_cs_ recorded in cells from knock-out cortex was not different than that seen in wild type cortex. To quantify the I-V curve shape we calculated the rectification index, which was expressed as a ratio of the slope of a linear fit between +30 and 40 mV to the linear fit between -70 and -60 mV [26]. The rectification indices of 5 wild type neurons, 5 TRPC1^-/-^ neurons and 3 TRPC5^-/-^ neurons (Fig 4B) were not significantly different.

## Discussion

These data lead us to conclude that I_cs_ is not a non-specific cationic current as previously proposed; rather, we suggest that it is mediated by delayed-rectifier K^+^ channels that are permeable to Cs^+^. Thus, the voltage dependence, the activation and deactivation kinetics and the pharmacological phenotype resemble other neuronal delayed rectifier K^+^ channels [27–29]. We used ion-sensitive dyes to directly test for Ca^2+^ and Na^+^ influx into the somatodendritic compartment while activating I_cs_ and found no evidence for such fluxes. Finally, we asked whether TRPC1 or TRPC5 participate in generating I_cs_ as has been hypothesized [4]. The kinetic and voltage-dependent characteristics of I_cs_ in TRPC1^-/-^ and TRPC5^-/-^ animals were not different from those recorded in WT.

The electrophysiological properties of I_cs_ recorded from murine layer 5 pyramidal cells are similar to those of other neuronal delayed rectifier voltage gated K^+^ channels. A study [28] in chick dorsal root ganglion (DRG) neurons demonstrates this resemblance; both currents activate when the membrane is depolarized above -20 mV, both have an activation time constant in the 5–25 ms range (shorter in more positive voltages) and a deactivation time in the 20–80 ms range at -90 to -60 mv [25–28]. Moreover, these delayed rectifier K^+^ channels from DRG neurons are permeable to Cs^+^ [27]. This is one of many reports highlighting the Cs^+^ permeation through delayed rectifier K^+^ channels [27,30–32]. Pharmacologically, I_cs_ was most sensitive to TEA, especially when used intracellularly. Our recordings show that I_cs_ amplitude was decreased by more than 75% when TEA was added to the recording electrode. Intracellular TEA has been reported to act as a selective delayed rectifier K^+^ channel blocker [25] and it is regularly used to differentiate K^+^ from TRP channels [33]. When added to the ringer, 40 mM TEA blocked approximately 50% of I_cs_, which is typical for several delayed rectifier K^+^ channels. Interestingly, relatively low TEA sensitivity has been reported for other Cs^+^ permeable forms of these channels [30,34]. I_cs_ was also sensitive to La^3+^, which is regularly used to block a wide range of TRP channels [35]. However, this compound cannot be considered as specific for TRP channels as they have been shown to be effective at blocking delayed rectifier K^+^ currents as well [36,37].

The presence of inward tail currents upon repolarization to -70 mV was originally taken [1] as an indication that I_cs_ is a non-specific cationic current, since the reversal potential is apparently more depolarized than E_K_. This, however, is more likely a reflection of the experimental recording conditions. In all of these experiments, as with those reported in [1], at least 3mM of K^+^ were included in the ACSF, and since the channels which generate I_cs_ pass Cs^+^ but have a higher permeability to K^+^, it is likely that tail current with a depolarized reversal potential is generated by inward K^+^ currents. The Goldman-Hodgkin-Katz equation predicts that the observed reversal potential of about -40 mV would be expected if the ratio of permeability for Cs^+^ vs. K^+^, *P*_Cs_ / *P*_K_, is around 0.11 (see methods). This permeation preference for K^+^ is similar to that reported for cesium-permeable delayed rectifier K^+^ channels in bullfrog neurons (*P*_Cs_ / *P*_K_ = 0.17) [27], though it is lower than that reported in embryonic chick dorsal root ganglion neurons (*P*_Cs_ / *P*_K_ = 0.25) [28].

TRP channels have been the focus of much interest in recent years, as evidence accumulates for their role in neuronal function. TRPCs mRNAs are widely expressed in many CNS tissues including cortex, hypothalamus, hippocampus, mid-brain and cerebellum [38–41]. Strübing et al. [4] showed that when the heteromer of TRPC1 and TRPC5 channel subunits are co-expressed they form a channel with characteristics different than those seen when the channels are expressed separately. In the developing mouse brain TRPC1 and TRPC5 mRNAs are strongly expressed in the cortex [42], suggesting that such a heteromer may play a major role in cortical neuronal function. However, such evidence is still lacking [43]. We found no evidence for direct involvement of TRPC1 and TRPC5 in the whole cell conductance of layer 5 neurons.

In summary, while our electrophysiological and imaging data in no way suggest that TRP channels are not present or that they don’t serve an important function; they do, however, indicate that these channels are not constitutively active in this cell type.

## References

[1] Alzheimer C. A novel voltage-dependent cation current in rat neocortical neurones. J Physiol. 1994;479: 199–205. 7528275 10.1113/jphysiol.1994.sp020288 PMC1155739

[2] Astman N, Gutnick MJ, Fleidervish I a. Persistent sodium current in layer 5 neocortical neurons is primarily generated in the proximal axon. J Neurosci. 2006;26: 3465–3473. 16571753 10.1523/JNEUROSCI.4907-05.2006 PMC6673860

[3] Fleidervish I a, Friedman a, Gutnick MJ. Slow inactivation of Na+ current and slow cumulative spike adaptation in mouse and guinea-pig neocortical neurones in slices. J Physiol. 1996;493: 83–97. 8735696 10.1113/jphysiol.1996.sp021366 PMC1158952

[4] Strübing C, Krapivinsky G, Krapivinsky L, Clapham DE. TRPC1 and TRPC5 form a novel cation channel in mammalian brain. Neuron. 2001;29: 645–655. 11301024 10.1016/s0896-6273(01)00240-9

[5] Cristino L, de Petrocellis L, Pryce G, Baker D, Guglielmotti V, Di Marzo V. Immunohistochemical localization of cannabinoid type 1 and vanilloid transient receptor potential vanilloid type 1 receptors in the mouse brain. Neuroscience. 2006;139: 1405–1415. 16603318 10.1016/j.neuroscience.2006.02.074

[6] Liapi A, Wood JN. Extensive co-localization and heteromultimer formation of the vanilloid receptor-like protein TRPV2 and the capsaicin receptor TRPV1 in the adult rat cerebral cortex. Eur J Neurosci. 2005;22: 825–834. 16115206 10.1111/j.1460-9568.2005.04270.x

[7] Minke B, Cook B. TRP channel proteins and signal transduction. Physiol Rev. 2002;82: 429–472. 11917094 10.1152/physrev.00001.2002

[8] Wei W-L, Sun H-S, Olah ME, Sun X, Czerwinska E, Czerwinski W, et al TRPM7 channels in hippocampal neurons detect levels of extracellular divalent cations. Proc Natl Acad Sci U S A. 2007;104: 16323–16328. 17913893 10.1073/pnas.0701149104 PMC2042205

[9] Sun H, Jackson MF, Martin LJ, Jansen K, Teves L, Cui H, et al Suppression of hippocampal TRPM7 protein prevents delayed neuronal death in brain ischemia Nat Neurosci. Nature Publishing Group; 2009;12: 1300–1307. 10.1038/nn.2395 19734892

[10] Aarts M, Iihara K, Wei W-L, Xiong Z-G, Arundine M, Cerwinski W, et al A key role for TRPM7 channels in anoxic neuronal death. Cell. 2003;115: 863–877. 14697204 10.1016/s0092-8674(03)01017-1

[11] Peters M, Trembovler V, Alexandrovich A, Parnas M, Birnbaumer L, Minke B, et al Carvacrol Together With TRPC1 Elimination Improve Functional Recovery After Traumatic Brain Injury in Mice. Journal of Neurotrauma. 2012;29:2831–2834. 10.1089/neu.2012.2575 22994850 PMC3521132

[12] Hamill OP, Marty A, Neher E, Sakmann B, Sigworth FJ. Improved patch-clamp techniques for high-resolution current recording from cells and cell-free membrane patches Pflugers Arch Eur J Physiol. Springer; 1981;391: 85–100. 6270629 10.1007/BF00656997

[13] Blanton MG, Lo Turco JJ, Kriegstein AR. Whole cell recording from neurons in slices of reptilian and mammalian cerebral cortex. J Neurosci Methods. 1989;30: 203–210. 2607782 10.1016/0165-0270(89)90131-3

[14] Stuart GJ, Dodt HU, Sakmann B. Patch-clamp recordings from the soma and dendrites of neurons in brain slices using infrared video microscopy Pflugers Arch Eur J Physiol. Springer; 1993;423: 511–518. 8351200 10.1007/BF00374949

[15] Haas HL, Schaerer B, Vosmansky M. A simple perfusion chamber for the study of nervous tissue slices in vitro. J Neurosci Methods. 1979;1: 323–325. 544974 10.1016/0165-0270(79)90021-9

[16] Fleidervish I a, Lasser-Ross N, Gutnick MJ, Ross WN. Na^+^ imaging reveals little difference in action potential-evoked Na^+^ influx between axon and soma Nat Neurosci. Nature Publishing Group; 2010;13: 852–60. 10.1038/nn.2574 20543843 PMC3102307

[17] Grienberger C, Konnerth A. Imaging Calcium in Neurons. Neuron. 2012 pp. 862–885. 10.1016/j.neuron.2012.02.011 22405199

[18] Ramsey IS, Delling M, Clapham DE. An introduction to TRP channels. Annu Rev Physiol. 2006;68: 619–647. 16460286 10.1146/annurev.physiol.68.040204.100431

[19] Merritt JE, Armstrong WP, Benham CD, Hallam TJ, Jacob R, Jaxa-Chamiec A, et al SK&F 96365, a novel inhibitor of receptor-mediated calcium entry. Biochem J. 1990;271: 515–522. 2173565 10.1042/bj2710515 PMC1149585

[20] Rychkov G, Barritt GJ. TRPC1 Ca2+-permeable channels in animal cells. Handb Exp Pharmacol. 2007;179: 23–52. 17217049 10.1007/978-3-540-34891-7_2

[21] Kiselyov K, Xu X, Mozhayeva G, Kuo T, Pessah I, Mignery G, et al Functional interaction between InsP3 receptors and store-operated Htrp3 channels. Nature. 1998;396: 478–482. 9853757 10.1038/24890

[22] Delmas P, Wanaverbecq N, Abogadie FC, Mistry M, Brown DA. Signaling microdomains define the specificity of receptor-mediated InsP3 pathways in neurons. Neuron. 2002;34: 209–220. 11970863 10.1016/s0896-6273(02)00641-4

[23] Minke B, Cook B. TRP channel proteins and signal transduction. Physiol Rev. 2002; 429–472. 11917094 10.1152/physrev.00001.2002

[24] Parnas M. Peters M. Minke B. 6.4 Biophysics of TRP channels In: Edward H.E., editor. Comprehensive Biophysics. Elsevier: Amsterdam; 2012 pp. 68–107.

[25] Hille B. The selective inhibition of delayed potassium currents in nerve by tetraethylammonium ion. J Gen Physiol. 1967; 50:1287–1302. 6033586 10.1085/jgp.50.5.1287 PMC2225709

[26] Briand L a, Kimmey B a, Ortinski PI, Huganir RL, Pierce RC. Disruption of glutamate receptor-interacting protein in nucleus accumbens enhances vulnerability to cocaine relapse. Neuropsychopharmacology. 2014;39: 759–769. 10.1038/npp.2013.265 24126453 PMC3895254

[27] Block BM, Jones SW. Delayed rectifier current of bullfrog sympathetic neurons: ion-ion competition, asymmetrical block and effects of ions on gating. J Physiol. 1997;499: 403–416. 9080370 10.1113/jphysiol.1997.sp021937 PMC1159315

[28] Trequattrini C, Petris A, Franciolini F. Characterization of a neuronal delayed rectifier K current permeant to Cs and blocked by verapamil. J Membr Biol. 1996;154: 143–153. 8929288 10.1007/s002329900139

[29] Klemic KG, Durand DM, Jones SW. Activation kinetics of the delayed rectifier potassium current of bullfrog sympathetic neurons. J Neurophysiol. 1998;79: 2345–2357. 9582210 10.1152/jn.1998.79.5.2345

[30] Rüsch A, Eatock RA. A delayed rectifier conductance in type I hair cells of the mouse utricle. J Neurophysiol. 1996;76: 995–1004. 8871214 10.1152/jn.1996.76.2.995

[31] Rennie KJ, Correia MJ. Effects of cationic substitutions on delayed rectifier current in type I vestibular hair cells. J Membr Biol. 2000;173: 139–148. 10630929 10.1007/s002320001015

[32] Wigmore MA, Lacey MG. A Kv3-like persistent, outwardly rectifying, Cs+-permeable, K+ current in rat subthalamic nucleus neurones. J Physiol. 2000;527: 493–506. 10990536 10.1111/j.1469-7793.2000.t01-1-00493.x PMC2270091

[33] Hardie RC, Minke B. The trp gene is essential for a light-activated Ca2+ channel in Drosophila photoreceptors. Neuron. 1992;8: 643–651. 1314617 10.1016/0896-6273(92)90086-s

[34] Rennie KJ, Correia MJ. Potassium currents in mammalian and avian isolated type I semicircular canal hair cells. J Neurophysiol. 1994;71: 317–329. 8158233 10.1152/jn.1994.71.1.317

[35] Ramsey IS, Delling M, Clapham DE. An introduction to TRP channels. Annu Rev Physiol. 2006;68: 619–647. 16460286 10.1146/annurev.physiol.68.040204.100431

[36] Sanguinetti M. C., and Jurkiewicz N. K.. 1990b Lanthanum blocks a specific component of IK and screens membrane surface charge in cardiac cells. American Journal of Physiology. 259:H1881–889. 2260712 10.1152/ajpheart.1990.259.6.H1881

[37] Tytgat J, Daenens P. Effect of lanthanum on voltage-dependent gating of a cloned mammalian neuronal potassium channel. Brain Res. Elsevier; 1997;749: 232–237. 10.1016/S0006-8993(96)01171-7 9138723

[38] Kunert-Keil C, Bisping F, Krüger J, Brinkmeier H. Tissue-specific expression of TRP channel genes in the mouse and its variation in three different mouse strains. BMC Genomics. 2006;7: 159 16787531 10.1186/1471-2164-7-159 PMC1557673

[39] Riccio A, Medhurst AD, Mattei C, Kelsell RE, Calver AR, Randall AD, et al mRNA distribution analysis of human TRPC family in CNS and peripheral tissues. Brain Res Mol Brain Res. 2002;109: 95–104 12531519 10.1016/s0169-328x(02)00527-2

[40] Zechel S, Werner S, von Bohlen Und Halbach O. Distribution of TRPC4 in developing and adult murine brain. Cell Tissue Res. 2007;328: 651–656. 17345099 10.1007/s00441-007-0388-4

[41] Voronova IP, Tuzhikova A a, Kozyreva T V. Gene expression of thermosensitive TRP ion channels in the rat brain structures: Effect of adaptation to cold. J Therm Biol. Elsevier; 2013;38: 300–304.

[42] Boisseau S, Kunert-Keil C, Lucke S, Bouron A. Heterogeneous distribution of TRPC proteins in the embryonic cortex. Histochem Cell Biol. 2009;131: 355–363. 10.1007/s00418-008-0532-6 18989690

[43] Phelan KD, Shwe UT, Abramowitz J, Wu H, Rhee SW, Howell MD, et al Canonical transient receptor channel 5 (TRPC5) and TRPC1/4 contribute to seizure and excitotoxicity by distinct cellular mechanisms. Mol Pharmacol. 2013;83: 429–38. 10.1124/mol.112.082271 23188715 PMC3558807

